# Extracellular vesicle-based drug delivery in pediatric immune thrombocytopenia

**DOI:** 10.3389/fimmu.2026.1951209

**Published:** 2026-10-23

**Authors:** Oriana Di Domenico, Maria Maddalena Marrapodi, Giuseppe Di Feo, Lucia Argenziano, Francesca Rossi, Alessandra Di Paola

**Affiliations:** 1 Department of Woman, Child and General and Specialist Surgery, University of Campania “Luigi Vanvitelli”, Naples, Italy; 2 Department of Life Sciences, Health and Health Professions, Link Campus University, Rome, Italy

**Keywords:** cell-free therapy, drug delivery, extracellular vesicles, immune thrombocytopenia, immunomodulation, mesenchymal stromal cells

## Abstract

Pediatric immune thrombocytopenia (ITP) is an acquired immune-mediated disorder triggered by autoantibody production directed against glycoproteins on the platelet surface, leading to platelet destruction in the spleen and impaired megakaryopoiesis in the bone marrow. The immune response is further compromised by a dysregulated inflammatory environment involving T-cell proliferation, pro-inflammatory macrophage polarization and defective immunomodulatory function of mesenchymal stromal cells (MSCs). Although first-line therapies such as intravenous immunoglobulin G and corticosteroids remain beneficial, they are also associated with relevant toxicities and do not fully address the systemic nature of the disease in pediatric patients. Extracellular vesicles (EVs) as cellular mediators secreted by all kinds of cells are currently under investigation as promising therapeutic nanocarriers. MSC-derived extracellular vesicles (MSC-EVs) in particular, have emerged as therapeutic candidates due to their low immunogenicity, high biocompatibility, and intrinsic ability to transport cargo capable of modulating the immune response. MSC-EVs may simultaneously regulate T-cell balance, influence macrophage polarization, support megakaryopoiesis, and deliver regulatory molecules to specific targets. Bioengineering strategies involving cargo loading and surface functionalization may further improve their therapeutic potential and homing capabilities. Although key barriers to clinical translation remain, MSC-EVs represent a promising platform for a precision and cell-free approach in pediatric ITP. Although direct pediatric-specific evidence remains limited, in this review we provide a comprehensive overview of the biological properties, immunomodulatory mechanisms, and translational challenges of MSC-EVs, comparing their advantages against conventional pharmacological approaches and other available synthetic nanocarriers.

## 1 Introduction

Immune thrombocytopenia (ITP) is an acquired immune-mediated disorder and the most common cause of thrombocytopenia in pediatric patients (1). It is characterized by a platelet count of less than 100 × 10^9^/L occurring in children between the ages of 1 and 18 years, resulting from autoantibody-mediated platelet destruction and impaired platelet production (2). These autoantibodies are directed against platelet glycoproteins (GP) IIb/IIIa or GPIb/IX, which results in engaging of both adaptive and innate immunity mechanisms (3).

The pathogenesis of ITP is multifaceted and involves the loss of immune tolerance to platelet and megakaryocyte antigens, expansion of autoreactive T and B lymphocytes, enhanced macrophage-mediated platelet phagocytosis, and suppressed megakaryopoiesis in the bone marrow, leading to a variety of clinical manifestations primarily involving coagulation deficits. Furthermore, dysregulation of the T helper (Th) 1/Th2 and Th17/regulatory T cell (Treg) balances contributes to the establishment of a pro-inflammatory environment that sustains autoimmune platelet destruction (4). **Figure 1** summarizes the key factors leading to the pathogenesis of pediatric ITP.

**Figure 1:**
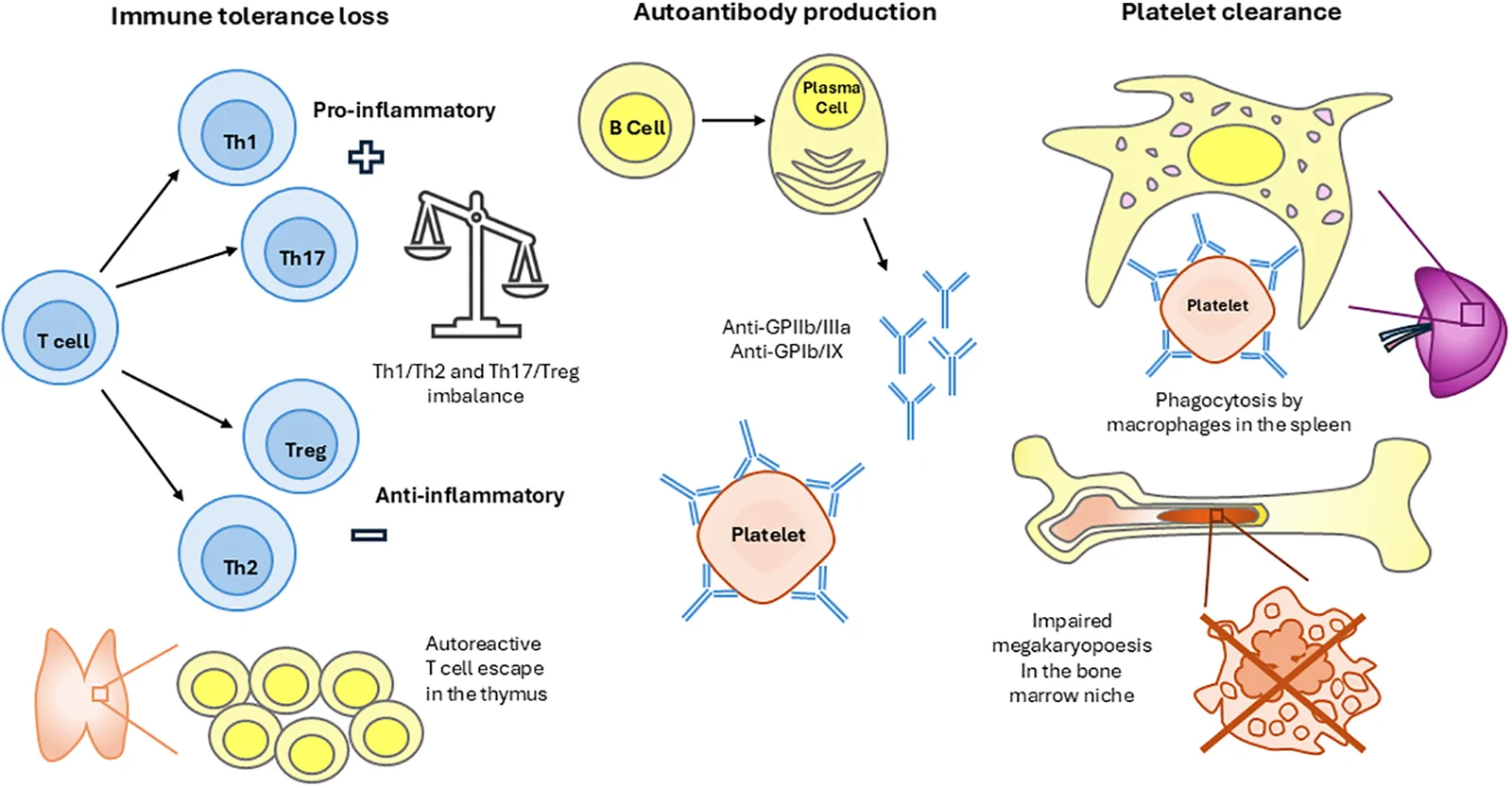
Schematic overview of the pathogenesis of pediatric ITP. The loss of immune tolerance leads to the escape of autoreactive T cells from thymic negative selection. These T cells, once in the periphery, provide help to autoreactive B cells specific for platelet glycoproteins GPIIb/IIIa and GPIb/IX, promoting their differentiation into antibody-secreting plasma cells. Resulting autoantibodies trigger peripheral platelet clearance via splenic macrophage phagocytosis and complement activation. Simultaneously, the autoimmune response is sustained through the production of pro-inflammatory cytokine, further exacerbating the T-cell subsets imbalance. Supporting and enhancing macrophage polarization toward an M1 pro-inflammatory phenotype and impairing megakaryopoiesis within the bone marrow niche.

Schifferli et al. highlighted the issue of misdiagnosed thrombocytopenia in children and adolescents, underscoring the importance of accurate classification (5). Broadly, ITP is clustered by the length of the disease and is classified as newly diagnosed or persistent, within 3 months, or between 3 to 12 months from diagnosis respectively, and lastly as chronic when the disorder lasts for more than 12 months (6). All forms of ITP have overlapping pathophysiology and exhibit similar signs and clinical symptoms, nevertheless, classification and prior identification of the disease are fundamental to dictate the following treatments and how the disease should be clinically managed (7).

Regarding clinical manifestations, they may vary widely depending on the patient and in particular, they range from asymptomatic or mild bruising and petechiae, to severe potentially life-threatening haemorrhages (8).

ITP presents a good prognosis for pediatric patients, where more than 70% of cases experience spontaneous recovery within six months of diagnosis, but up to 30% develop chronic ITP (9). A higher risk of chronicity is observed as the age of onset or diagnosis increases, reaching a peak at around over 10 years (10, 11).

Overall, ITP as a disease significantly impacts the quality of life (QoL) of the patients, primarily due to restrictions in daily recreational and sports activities, resulting in social isolation. In recent years, increased attention to assessing the QoL of ITP patients has highlighted how thrombocytopenia negatively affects not only mood and state of mind, but also physical energy and cognitive performance (e.g. chronic fatigue syndrome) in a significant number of pediatric patients (12).

Regarding treatment options for ITP, it is reserved in patients with severe symptoms, such as significant bleeding, therefore manifestations that impact the QoL of both the child and family. In fact, treatment decisions are based primarily on bleeding and clinical manifestation severity, not on the platelet count alone. As previously stated, the majority of ITP patients do not present any symptoms, therefore they do not require any pharmacologic treatment (13). According to the latest therapy plan proposed by the “Associazione Italiana Ematologia Oncologia Pediatrica” (AIEOP) committee in 2024, the first line of therapy involves the use of intravenous immunoglobulin G (IVIG) or high-dose corticosteroids, such as prednisone or intravenous methylprednisolone, administered orally or parenterally. Second-line agents are indicated for persistent or chronic ITP with significant bleeding, impaired QoL, or dependence on first-line treatments, and encompass the use of several different drugs (10, 13). Thrombopoietin Receptor Agonists (TPO-RAs) like Eltrombopag, Avatrombopag and Romiplostim, are employed to stimulate platelet production and are administered orally (14, 15). Among other second-line agents, immunosuppressive agents such as Mycophenolate Mofetil (MMF), and Sirolimus in refractory cases are also established options (16–18). In the case in which all previous therapy plans were not successful, Rituximab, a monoclonal antibody against CD20, a glycoprotein expressed on the surface of B cells leading to a reversible B-cell depletion, represents another important second-line option to implement (19–21). Regarding patients who do not present any symptoms, absence or mild bleedings, observation is mandatory. In contrast, severe bleeding, life threatening situations or refractory cases may require combined therapies involving IVIG together with corticosteroids or platelets transfusions. Splenectomy is considered only in exceptional circumstances in pediatric patients and is strongly limited to children >5 years of age, after failure of available medical therapies, in chronic disease and when QoL is substantially impaired (13, 21).

Despite the availability of a plethora of different therapeutic approaches based on the needs of the patients, all of them present several limitations and serious adverse effects. Specifically, common complications of first-line therapies include gastrointestinal and renal toxicity, weight gain, sleep and behavioral disturbances, increased susceptibility to infections, thrombosis, hypertension, and hyperglycemia, particularly following prolonged treatment (22, 23).

Understanding the nuances of this condition and emerging therapeutic strategies may help in improving patient outcomes, therefore major attention has been given to the developing of novel therapeutic approaches in treating such a clinical variable disease. These limitations have highlighted the need to search for novel therapeutic approaches that can address the multifaceted pathophysiology of ITP while lessening the overall side effects (9). In this context, extracellular vesicles (EVs) have emerged as promising biological nanocarriers with inherent immunomodulatory properties.

EVs are particles delimited by a lipid bilayer membrane, variable in size ranging from nanometers to micrometers, that are released by all cell types and are incapable of self-replication (24). Nowadays, EVs are considered among the most promising candidates for therapeutic development, specifically due to some of their most prominent properties, such as low immunogenicity, biocompatibility, ability to cross biological membranes, therefore these abilities taken all together position them as versatile carriers for targeted drug delivery (25).

The primary aim of this review is to summarize and encompass the current knowledge and focus attention on the promising and novel therapeutic opportunities revolving around the implementation of EVs as a potential drug-delivery and free-cell therapeutic option in pediatric ITP. While other reviews have already addressed the therapeutic potential of EVs in ITP as a generic immunomodulatory platform, their scope has predominantly been confined to the adult population or to a broad description of MSC-EV biology and their therapeutic mechanisms. The present review builds upon prior literature focusing explicitly on pediatric ITP, contextualizing EVs-based therapies within the age-specific epidemiology and the functional impairment of MSCs in ITP patients resulting in ineffective autologous cell-based approaches and answering the primary question of whether and how extracellular vesicles, and MSC-derived EVs in particular, could be developed and integrated as drug-delivery systems for the treatment of pediatric ITP. Moreover, it provides a side-by-side comparison of MSC-EVs with alternative nanocarrier platforms, including liposomes, lipid nanoparticles (LNPs), platelet membrane-coated nanoparticles (PNPs), and whole MSCs therapy, essential to delineate the actual translational niche that EVs may occupy. Lastly, it discusses EVs engineering and cargo-loading strategies, an area that remains largely unexplored in previous ITP-specific literature, but that will likely determine the clinical future advancement of this platform.

## 2 Extracellular vesicles: biology and classification

Despite firstly being reported as merely a different way for disposing of debris and waste material in the cell, in recent years EVs have been the subject of much more scrutiny from the scientific community, gaining substantial interest for their promising and versatile implementation in various sectors (26). The first ever reported sighting and description of EVs was in 1946 by Chargaff and West observing particles derived from platelets in plasma, enhancing and promoting coagulation (27).

EVs are secreted in various degrees by all kinds of cells, moreover, they are segregated in different subcategories based primarily on biogenesis, size, cargos and function. According to the “Minimal information for studies of extracellular vesicles” (MISEV) 2023 guidelines, it is advised against using a specific nomenclature depending on different biogenesis of EVs or size, such as microvesicles or exosomes, whereas the overall classification of vesicles is recommended to be into: extracellular vesicles, synthetic vesicles, and artificial cell-derived vesicles (24). Despite the discouragement to adopt such nomenclature, nowadays the employment of different EVs’ names is still commonly based on the previous division (**Figure 2**). Nevertheless, following the MISEV2023 guidelines, in this review all types of vesicles will be collectively referred to as EVs.

**Figure 2:**
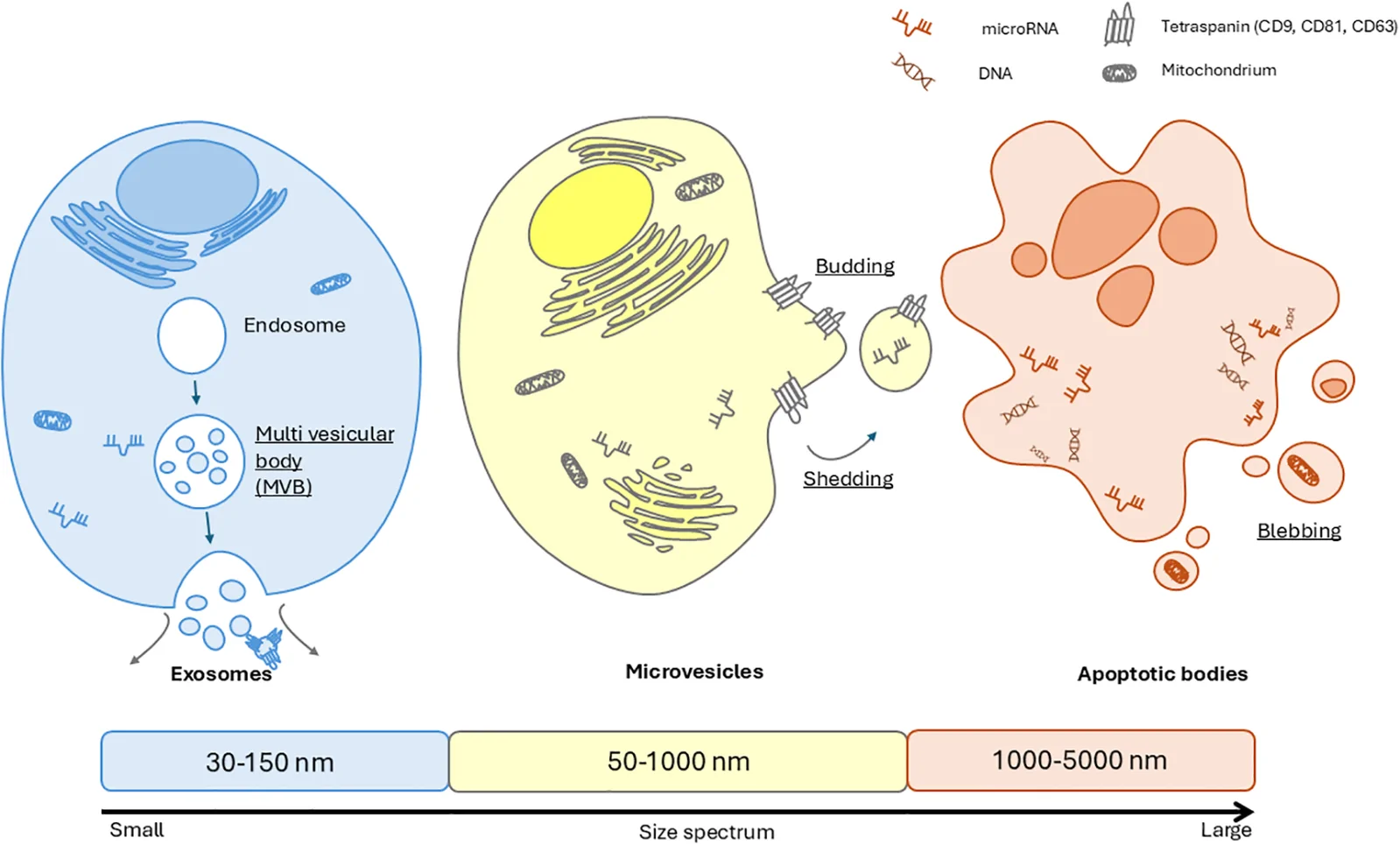
Historical classification and biogenesis of extracellular vesicles. EVs are traditionally divided into three subtypes based on biogenesis and size: exosomes (30–150 nm), originating from the endosomal pathway via multivesicular bodies (MVBs); microvesicles (50–1000 nm), generated by direct outward budding and shedding of the plasma membrane; and apoptotic bodies (1000–5000 nm), the largest subclass, formed through membrane blebbing during programmed cell death. This size-based classification, while widely used in literature and by the scientific community, it is not fully supported by current MISEV2023 guidelines which recommend the collective term extracellular vesicles due to the difficulty of isolation and classification.

EVs harbor an array of bioactive molecules, which would otherwise be degraded in the extracellular environment, thereby exhibiting a protective role for these molecules, which include proteins, lipids, and nucleic acids such as RNA (microRNA, mRNA, etc.) originating from their parental cells. Furthermore, it is important to point out that even within the same cell type, EV populations are heterogeneous. For instance, mesenchymal stromal cells (MSCs) may release distinct EVs differing in cargo, surface markers or size depending on variables such as culture conditions or donor availability, which concur in one of the major limitations of using EVs: manufacturing reproducibility (28).

EVs are well-known for their intrinsic stability, low immunogenicity, biocompatibility, extended half-life and enhanced ability to cross biological barriers (e.g. Blood Brain Barrier), reaching specific organs difficult to target by normal drugs or many synthetic particles. Furthermore, they physiologically already serve as mediators and carriers of several molecules whose role is pivotal in regulating intra and extracellular communication and might be bioengineered from parent cells, addressing the complex manufacturing challenges associated with the production of other delivery systems (29). Therefore, EVs play a fundamental role in physiological and pathological processes of the whole organism and may represent a novel cell-free therapeutic alternative, as drug and gene delivery systems, nutraceutical carriers, or biomarkers for liquid biopsies, disease diagnosis and prognosis (30).

### 2.1 Sources of EVs and their immunomodulatory functions

Notably, EVs have been extensively studied as pivotal effectors in regulating the equilibrium of the immune system and how it responds and reacts to stimuli for future therapeutic application (31). Recent studies have demonstrated that EVs, based on the conditions and the kind of cell of origin, perform diverse and essential roles in immune system response (32). EVs exercise their pivotal function through two major ways: directly affecting the target immune cell to activate downstream signals, or indirectly by transporting microRNAs (miRNAs). However, EVs production and action in immune responses modulation is convoluted and is further aggravated as EVs also act as intercellular signals, thereby they are constantly generated by all immune cells and tissue resident cells, rendering it difficult to describe the overall microenvironment (33).

In the context of different pathologies, EVs produced by B cells, T cells, Natural Killer (NK) cells, dendritic cells (DCs), macrophages, MSCs and neutrophils likely work in a coordinated fashion to provide positive and negative signals for inflammation, thus depending on the cell of origin, EVs may carry out different functions. Furthermore, surface ligands on EVs may be involved in regulating their uptake by specific target cells, further impacting immune response. EVs can transport cytokines to regulate other immune cells’ polarization and activity, and also, both MHCI- and MHCII-antigen complexes have been reported on EVs, which may be presented to T cells (34–36).

Beyond the transfer of MHC-antigen complex between cells by EVs, the latter may also induce additional signals to immune cells by transferring intact receptors to the plasma membrane of target cells (34). In particular, EVs from DCs and from B cells directly stimulate T cell proliferation and cytokine production *in vitro* (37). DCs are fundamental not only for their primary function as antigen presenting cell (APC), but they have been reported to secrete EVs for antigen presentation and adaptive response. On the other hand, EVs derived from T cells are generally involved in antiviral and antitumoral activity, and their production and secretion is enhanced by stimulation of T cell receptor (38, 39).

Other fundamental cells in managing innate immunity and immune responses in general are macrophages which play various different functions depending on the polarization and stimulant molecules in the environment. EVs derived from macrophages may have an inhibitory or stimulatory effect on immune functions depending on the polarization of the producer macrophage and type of cargo (40, 41).

B cells are the source of anti-platelet autoantibodies, being the main drivers of ITP pathogenesis, therefore in ITP, B cell-derived EVs carry pathogenic cargo including autoantibody-loaded immune complexes and pro-inflammatory miRNAs that further amplify the humoral immune response against platelets (31).

Understanding how EVs derived from different immune cell populations participate in immune regulation and dysregulation provides the framework for interpreting the pathogenic role of endogenous EVs in ITP and immune diseases in general, and the therapeutic potential of MSC-EVs discussed more in depth in the following sections.

### 2.2 MSC-derived EVs in ITP: mechanisms and therapeutic potential

Among the various cellular sources of EVs, those isolated from MSCs are the most studied nowadays (42).

MSCs are multipotent stem cells that have already been extensively studied in several diseases due to their ability to migrate to damaged tissues or organs in response to inflammatory stimuli. In fact, one of their main functions is to ensure the equilibrium of the immune system by acting as modulators of the immune response. MSCs may be isolated from various sources, including: bone marrow, umbilical cord, placenta, adipose tissue, and dental pulp, and have emerged as promising therapeutic candidates due to their unique biological properties in treating a wide range of inflammatory diseases (43). Furthermore, MSCs are known to interact with other immune cells and are implicated in both innate and adaptive immune response thanks to their role in regulating the surrounding microenvironment. In particular, MSCs and macrophages engage in a bidirectional crosstalk where MSCs drive macrophage polarization toward an anti-inflammatory M2 phenotype through the secretion of regulatory cytokines such as interleukin-10 (IL-10) and macrophages are able to modulate simultaneously MSCs’ secreted molecules by their produced stimuli. Conversely, MSCs can promote T-cell activation and, through IFN-γ and TNF-α production, support M1 pro-inflammatory polarization when necessary, regulate Th1 and Th2 balance, modulate the activity of NK cells, influence B-cell functions, thereby antibody production, and DCs maturation (44).

Regarding MSCs in ITP, keeping in mind that ITP pathogenesis involves alterations in both inflammation and immune response, and MSCs have been seen to be implicated in immune modulation, the role of MSCs in ITP has been extensively studied. Specifically, MSCs isolated from the bone marrow of pediatric patients affected by ITP have been reported to present compromised functions and activity, and interestingly, they exhibit a reduced proliferation capacity together with an increase in apoptosis, senescence, and functional impairments resulting in a lessening of their immunosuppressive and anti-inflammatory activities such as the inhibition of T-cell proliferation (45–47).

Recent evidence in a murine model also highlights that the activation of the HMGB1-TLR4 pathway in these cells leads to a marked disruption in macrophage polarization, further compromising the bone marrow niche. This evident MSCs dysfunction not only dysregulates the systemic immune response but also directly hinders megakaryopoiesis and platelet formation (48).

Because MSCs in ITP patients are functionally impaired, direct autologous cell therapy is often ineffective. EVs act as the primary means in the paracrine communication of MSCs, carrying the same immunomodulatory cargo but without the risks associated with injecting impaired or senescent cells (49). Consequently, restoring homeostasis through healthy, allogeneic MSC-derived products such as EVs has emerged as a superior strategy. The therapeutic effects originally attributed to whole MSCs are now understood to be largely mediated by their secretome, particularly the molecules encapsulated within EVs derived by MSCs (MSC-EVs) (44).

MSC-EVs are among the most feasible and interesting type of physiological cell-free therapeutical approach, thanks to their high yield and production capacity, widely different functions and broad clinical applications, thereby drugs based on MSC-EVs are the closest to achieve clinical translation and industrial production. Nevertheless, to translate these findings into clinical practice, the manufacture of MSC-EVs must be standardized under good manufacturing practice (GMP). Recent advances have moved toward reproducible and scalable high-yield production processes and by utilizing three dimensional (3D)-bioreactor systems and refined filtration techniques, it is now possible to generate large quantities of standardized MSC-EVs that maintain their immunomodulatory cargo, overcoming the limitations of traditional 2D culture methods (50).

Beyond their therapeutic application, MSC-EVs are gaining traction as novel diagnostic tools, possessing the potential to deliver immunomodulatory molecules while serving also as prognostic biomarkers. Specifically, transcriptomic analysis has identified specific EV-related genes that are differentially expressed in ITP patients, suggesting that EV-cargo profiles could serve as candidate biomarkers for disease monitoring and treatment response (51). Genes reported as altered in EVs isolated from ITP patients, appear to mediate the abnormal crosstalk between immune cells and megakaryocytes, positioning them as potential candidate biomarkers for diagnosing primary ITP and monitoring treatment (52).

Combining these diagnostic insights with the therapeutic potential of MSC-EVs could pave the way for a novel, personalized and cell-free approach to managing pediatric ITP.

## 3 Endogenous cargo and engineering strategies

The therapeutic efficacy of EVs is fundamentally determined by the molecules transported to the target and recipient cell. While endogenous cargo mediates the natural immunomodulatory effects of MSC-EVs, bioengineering allows these vesicles to be repurposed as precision drug delivery systems, thereby tailoring the cargo to the specific needs of the patients. In their natural state, MSC-EVs and EVs in general, carry a molecular signature derived from their parent cells which is crucial for homeostatic immune regulation.

The most well studied molecules transported in EVs are miRNAs, short non-coding RNAs which post-transcriptionally regulate gene expression, modulating processes such as proliferation, differentiation, apoptosis and metabolism (53). For instance in ITP, miRNAs are naturally packed in EVs to modulate immune cell response and polarization. In particular, miR-146a-5p and miR-199a-5p have been proposed as biomarkers for MSC-EVs in ITP, and are known to suppress pro-inflammatory signaling pathways (like NF-κB via IRAK1) and promote the transition of macrophages from the M1 to the M2 phenotype (pro-inflammatory to anti-inflammatory phenotype) (54–56). Furthermore, these same miRNAs have been investigated more broadly in relation to the classical T cell imbalances (Th1/Th2 and Th17/Treg) distinctive of ITP pathology and have been seen to be implicated in ameliorating Th17/Treg ratio (57, 58).

EVs may also contain proteins, lipids, or other key molecules which modulate immune response, such as Programmed Death Ligand-1 (PD-L1), galectin-1 and TGF-β, which are described as regulators of peripheral tolerance that actively suppress auto-reactive cells and induce immune tolerance (59).

Another miRNA currently under investigation related to ITP pathogenesis is miR-155-5p, already studied for its role in other immune related disorders (60, 61). Although miR-155-5p has not been studied yet in relation to EVs, it has been under examination in regards to ITP pathogenesis in general. In fact, miR-155-5p is shown as upregulated in PBMCs isolated from ITP patients and has been reported to regulate macrophage M2 polarization through PD1/PD-L1 axis, restoring physiological expression level of SOCS1, commonly downregulated in ITP (62).

However, the pathogenic role of endogenous EVs in ITP must also be acknowledged. Plasma-derived EVs from ITP patients have been shown to modulate platelet production and impair megakaryocyte apoptosis via Bcl-xL/caspase signaling, highlighting the dual nature of EVs, both as disease mediators and therapeutic vehicles (63). On the other hand, it has also been reported that EVs isolated from plasma extracted from ITP patients present elevated levels of miR-363-3p, which decrease Tregs ability to suppress Th1 cells through the downregulation of ARID3A, SPI1, and TBX21 expression, thereby exacerbating the immune dysfunction recorded in ITP (64).

Therefore, it is imperative to characterize the molecular cargo of therapeutic EVs to ensure they carry immunomodulatory rather than pathogenic payloads.

**Table 1** presents an overview of the endogenous MSC-EVs cargo and their immunomodulatory mechanisms in pediatric autoimmune diseases not limited to pediatric ITP but including juvenile systemic lupus erythematosus (SLE) and type 1 diabetes. Juvenile SLE and type 1 diabetes were selected as comparator diseases because they are pediatric autoimmune conditions sharing key pathogenic features with ITP, including T-cell imbalance and autoantibody-mediated effector mechanisms, thereby providing transferable findings for MSC-EVs immunomodulation. For each study, the table reports the disease model, the EVs source, the cargo examined and the major findings. Notably, no study to date has directly evaluated MSC-EVs in pediatric ITP, a gap further discussed in Section 7.1 fully dedicated to EVs limitations.

**Table 1:** Overview of endogenous MSC-EVs cargo and their immunomodulatory mechanisms in pediatric autoimmune diseases.

Disease	EVs source	Endogenous cargo	Model of study	Major findings	References	Evidence level
Pediatric ITP	Bone marrow MSC-EVs	miR-146a-5p	*In vivo* murine model	Targets IRAK1, regulates Th17/Treg balance via NF-kB suppression, promotes M2 macrophage switch	(57)	Indirect
Pediatric ITP	Adipose MSC-EVs, Plasma EVs	miR-199a-5p	*In vivo* murine model, adult ITP patients	Inhibits Th17 differentiation, suppresses pro-inflammatory signaling, proposed diagnostic biomarker	(54, 58)	Indirect
Juvenile SLE	Umbilical cord MSC-EVs (exosomes)	miR-155	*In vitro* B-cells from patients	miR-155 target SHIP-1 in B cells exerting immunomodulatory effects	(111)	Indirect
Type 1 diabetes	Gingival MSC-EVs (exosomes)	miR-23a-3p	*In vivo* autoimmune insulitis model	Targets IL-6R, suppresses JAK1/STAT3 signaling, expands Tregs	(112)	Indirect
Overall MSC-EVs	MSC-EVs (microvesicles)	PD-L1, galectin-1, membrane-bound TGF-β	*In vivo* autoimmune model	Tolerogenic proteins suppresses autoreactive lymphocyte proliferation, promotes IL-10 and TGF-β secretion	(59)	Indirect

The table summarizes key cargo molecules identified in MSC-EVs across different disease contexts, among ITP, juvenile systemic lupus erythematosus (SLE) and type 1 diabetes. Each entry reports the disease, model of study, EVs source, cargo, major findings and evidence level regarding its implementation in the human pediatric ITP context (Indirect or Direct).

### 3.1 Loading methods and surface modifications

To overcome the limited array of physiological available molecules already contained in EVs and to deliver exogenous drugs by this novel means, bioengineering strategies are currently being investigated and employed, to modify either the vesicle surface or the internal cargo (65). Engineering of EVs may be conducted in three different main ways: endogenous loading, exogenous loading and surface functionalization.

Firstly, loading therapeutic molecules into EVs, thereby modifying their endogenous cargo, can be achieved prior-isolation, or post-isolation of EVs, respectively via genetic engineering or physical/chemical methods (66).

Endogenous loading is defined as a strategy involving the loading of therapeutic cargo into the parent MSC prior to EVs collection, thus allowing the therapeutic miRNAs or proteins to be naturally sorted into EVs during biogenesis (67). Methods such as transfection and electroporation are the most common approaches, although only a fraction of the total EV population may successfully incorporate the therapeutic payload while maintaining the correct morphology and surface markers (68). For instance, Li et al. (2021) reported that adipose-derived MSCs transfected with miR-199a-5p oncomir resulting in its overexpression, produced EVs with enhanced immunomodulatory capacity alleviating ITP in a mouse model (58). Endogenous loading leverages the cell’s natural sorting machinery with the aim of yielding therapeutic cargos, conversely to exogenous loading techniques.

Specifically, exogenous loading is a method which enables cargo insertion directly into EVs, after their isolation without compromising the integrity of the whole cell (69). The most common methods employed are electroporation, sonication and freeze-thaw cycles. Electroporation uses electrical pulses to transiently permeabilize the EV membrane, allowing entry of the therapeutic molecules such as: siRNA, miRNA, or small molecules, whereas sonication disrupts the lipid bilayer temporarily for cargo loading. Broadly, all previously mentioned methods may result in surface protein denaturation and disruption of membrane integrity which are major limitations, the choice of method depends on cargo structure and type of molecule, loading efficiency, and downstream application (70). Nowadays, novel methods are always under investigation and saponin-mediated permeabilization has shown promise as it has been reported to improve cargo retention while preserving EVs surface marker integrity (71).

Regarding surface modifications and functionalization strategies, the aim is to enable targeted delivery to specific tissues relevant to the pathology. For ITP, targeting EVs to the bone marrow niche could enhance megakaryocyte rescue, while splenic targeting could modulate macrophage polarization at the site of platelet clearance. For example, several approaches have been explored, including peptide display, antibody conjugation via click chemistry, and genetic fusion of targeting ligands to tetraspanins such as CD63 (72). These strategies have been shown to significantly improve tissue-specific biodistribution and therapeutic efficacy compared to unmodified EVs, which tend to accumulate primarily in the liver and spleen following systemic administration (73).

## 4 From MSC cell therapy to MSC-EV drug delivery

In recent years, although the clinical application of MSCs has become increasingly widespread for the treatment of different pathologies, its pertinence in ITP is limited by the functional impairment of patient-derived MSCs and by the risks associated with whole cell infusion. MSCs owe their low immunogenicity to a minimal expression of MHC I molecules and the absence of MHC II and co-stimulatory molecules such as CD80, CD86 and CD40, which prevents effective lymphocyte activation and underlies their immunomodulatory action through direct cell-to-cell contact, secretion of soluble factors, and metabolic regulation (74).

Ryoncil (remestemcel-L) represents the first MSC-based therapy to receive approval by the FDA in pediatric setting, having been evaluated for graft-versus-host disease and it provides a precedent for MSCs-derived products in children (75).

Although studies and applications of MSCs-based therapy in ITP are reported as effective, they are all based on a cohort of study of adult patients and no clinical application in pediatric areas has been reported to our knowledge (76). Recent research has shown that 3D culture methods have many advantages over conventional culture with respect to MSCs’ secretion, homing and immunogenicity in comparison to bidimensional methods (77). On the other hand, intravenously administered MSCs may possess procoagulant activity due to the expression of tissue factor and phosphatidylserine, which increase the risk of thrombotic events (78). Regarding patients affected by ITP, these coagulation-related properties are of direct clinical relevance. Therefore, transition from whole-cell MSCs therapy to MSCs-EVs addresses several of the limitations inherent to cell-therapy, resulting in replicating the paracrine immunomodulatory functions of their cells of origin albeit removing the concurrent risks associated with intact cell infusion (79).

As drug-delivery platforms, MSC-EVs combine the paracrine cargo of their parental cells with a membrane optimized for circulation and targeting. The EV membrane is enriched in tetraspanins (CD63, CD81, CD9) and signals which protect them and extend their half-life in circulation (like CD47), and aid in evading degradation. The membrane bilayer itself contains bioactive lipids that can further stabilize the anti-inflammatory microenvironment of the bone marrow niche (49).

MSC-EVs may exert their immunomodulatory effects through various pathways such as restoring T-cell homeostasis by shifting the Th1/Th2 balance toward anti-inflammatory Th2 and restoring the Th17/Treg ratio, facilitating macrophage polarization toward an M2 phenotype and improving the bone marrow microenvironment to support megakaryopoiesis and platelet production (**Figure 3**).

**Figure 3:**
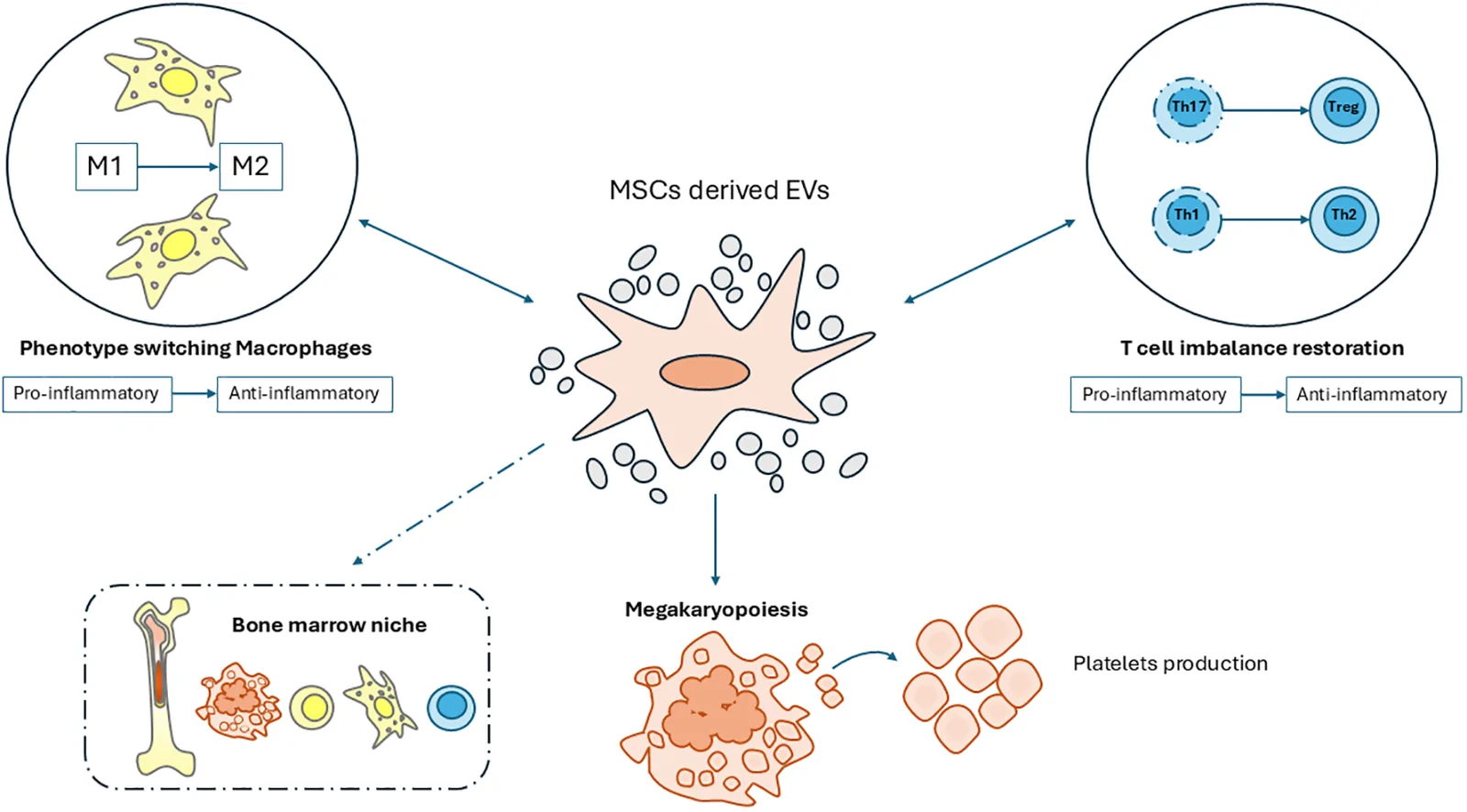
Proposed therapeutic mechanism of MSCs derived EVs in pediatric ITP. A schematic representation of how MSC-EVs as a cell-free therapy might address the systemic dysregulation underlying pediatric ITP. MSC-EVs may exert their immunomodulatory effects through multiple parallel pathways such as restoring T-cell homeostasis by shifting the Th1/Th2 balance toward anti-inflammatory Th2 and promoting the expansion of regulatory T cells over pro-inflammatory Th17 subpopulation; facilitating macrophage polarization from the pro-inflammatory M1 toward the anti-inflammatory M2 phenotype with subsequent production of anti-inflammatory molecules such as IL-10 and TGF-β; and improving the bone marrow microenvironment to support megakaryopoiesis and platelet production.

## 5 Comparison of therapeutic platforms for pediatric ITP

To define the translational niche of EVs in pediatric ITP, we briefly discuss the synthetic nanocarriers already implemented for drug delivery as benchmarks, allowing an explicit comparison between platforms. Nowadays, the interest in the development of synthetic nanocarriers for ITP therapy is ongoing, yet each individual platform carries inherent limitations. In previous years, an emerging interest in novel therapeutic approaches led to the development of liposome-based therapies.

Novel therapeutic application in ITP is the development of PNPs, which act as decoys to absorb pathogenic anti-platelet antibodies. Although this approach has been reported as functional in a murine model of antibody-induced thrombocytopenia, it is inherently single-target based on the neutralization of the auto-antibody and does not address the broader immune dysregulation (T-cell imbalance, MSCs dysfunction, impaired megakaryopoiesis) that characterizes pediatric ITP (80). Nevertheless, since this proof-of-concept the platform has substantially evolved. Recent reviews document the extensive role of PNPs, their immunocompatibility, prolonged circulation and their implementation into targeted therapies for cancer, immune-mediated, inflammatory diseases and cardiovascular disorders (81). In the latter setting specifically, PNPs exploit the adhesion molecules retained on the membrane, such as GPIIb/IIIa and P-selectin ligands, to achieve pathological-site homing and retention through specific molecular recognition, a strategy now applied to thrombus-targeted thrombolysis (82). Nevertheless, in the ITP context the decoy concept remains anchored to the original murine proof-of-concept (80), and the approach remains inherently single-target, based on antibody neutralization rather than on the correction of the broader immune dysregulation documented in pediatric ITP patients.

In the past and still nowadays, synthetic lipid-based materials such as liposomes and lipid nanoparticles have been integrated and used for drug delivery. Notably, several famous drugs use a lipid base system to be delivered, for example mRNA based COVID-19 vaccines by Moderna and Pfizer-BioNTech (83). Despite the innovative use of such implements, liposomes and lipid nanoparticles such as these face inherent limitations in relation to clearance, accumulation, immune responses, and although together with EVs they share a phospholipid bilayer structure, their biological properties diverge substantially (84). Liposomes’ synthetic lipid composition triggers immune recognition, particularly when PEGylated, leading to accelerated blood clearance upon repeated administration (85). Moreover, they lack the surface receptor that regulates cell-specific homing, therefore rendering active targeting difficult and dependent on the strategies of delivery implemented (86).

Despite liposomes having been explored in preclinical ITP models, none have yet reached clinical translation. A clinical study of Doxil in the treatment of patients with ITP (NCT00107913) is the only clinical study on pediatric ITP which employs liposomes, nevertheless the study’s status is unknown and is quite dated. Notably, another study reported that liposome-encapsulated clodronate depletes splenic macrophages in a murine ITP model, reducing platelet clearance resulting in ameliorating the disease. However, this strategy relies on broad macrophage elimination, compromising innate immune defense, a risk in pediatric populations where infection vulnerability is higher (87). On the other hand, Deng and Balthasar developed antibody-coated liposomes as decoys to neutralize circulating anti-platelet antibodies that, despite being a promising strategy, it is a single-target approach that does not address the complex pathophysiology of ITP (88). Both strategies, despite their ingenuity, have failed to advance beyond the preclinical stage over the past two decades. In contrast, MSCs-derived EVs offer a multitarget therapeutic platform that simultaneously modulates T-cell balance, suppresses autoantibody production, promotes megakaryocyte maturation and restores the bone marrow niche, without the drawback of macrophages depletion leading to a compromised immune response (**Table 2**).

**Table 2:** Comparative evaluation of therapeutic platforms for pediatric ITP, including PNPs, LNPs, MSC-EVs and MSC cell-therapy.

	PNPs	Liposomes/LNPs	MSC-EVs	MSC Cell-Therapy
Biocompatibility	Moderate	Moderate	High	High
Targeting	Requires surface ligand conjugation (folate)	Passive targeting, active targeting (PEGylated)	Inherent tropism via surface adhesion molecules (integrins, tetraspanins)	Homing to injury sites via SDF-1/CXCR4 axis, poor specificity in systemic delivery
Cargo capacity	Small molecules or nucleic acids	Small molecules or nucleic acids	Endogenous miRNAs, proteins, lipids, metabolites, multi-target mechanism	Whole-cell secretome and paracrine effects, variable cargo depending on cell source and passage
Immune modulation	Limited, can be engineered	Moderate, some lipid formulations activate innate immunity; adjuvants	High, intrinsic immunomodulatory activity	Moderate, via secreted factors, senescent MSCs may lose their capabilities or acquire pro-inflammatory phenotype
Stability	Degradable, limited shelf life	Depending on storage	Stable at -80 °C, lyophilization with trehalose preserves integrity, less stable at 4 °C	Cryopreservation is feasible but viability loss upon thawing, batch-to-batch variability in cell number and viability
Scalability & Manufacturing	Established GMP	Established GMP	Require large-scale culture, EV isolation (UC, SEC, TFF), characterization (NTA, microscopy, Western Blot), no fully validated GMP protocol yet	Established GMP, donor expanding/harvesting variability
Safety concerns	Potential cytotoxicity	Complement reactions, neurotoxicity	Minimal toxicity reported (procoagulant activity)	Procoagulant risk (tissue factor expression), tumorigenicity concern with prolonged culture, donor-site morbidity
Clinical Application	Well-defined	Well-defined, various approved products (e.g. COVID-19 vaccines, Vyxeos)	Preclinical/early clinical trials still underway, no approved EVs therapy yet	Well-defined, no therapy approved for autoimmune disease, however various approved in other field (e.g. Temcell)

The table compares biocompatibility, targeting, cargo capacity, immune modulation, stability, scalability, safety concerns and clinical translation of the abovementioned different therapies. Platelet membrane-coated nanoparticles (PNPs), lipid nanoparticles (LNPs), extracellular vesicles derived from Mesenchymal stromal cells (MSC-EVs).

Notably, liposome-based approaches have also achieved regulatory approval and clinical success in other pediatric haematological diseases, for instance, Vyxeos (CPX-351), a liposomal daunorubicin-cytarabine formulation approved by the FDA for newly diagnosed therapy-related Acute Myeloid Leukemia (AML) and AML in adults and pediatric patients aged one year and older, and Marqibo, a liposomal vincristine formulation evaluated in pediatric Philadelphia chromosome-negative acute lymphoblastic leukemia (89, 90). These clinical successes demonstrate that liposomal drug delivery is feasible in children, however, both agents are cytotoxic carriers designed for malignant targets, whereas ITP requires immunomodulatory capabilities and precision that may be acquired via engineering of different nanocarriers.

The use of EVs, which show among the advantages also the same ones related to lipid-based-molecules, overcome some of the previously mentioned issues. Specifically, EVs inherit the full repertoire of parental-cell membrane proteins, adhesion molecules and receptor ligands including CD47 and other surface signals which confer prolonged circulation times and enabling tissue-specific targeting, pivotal for direct homing to inflamed bone marrow and tissues without the need for synthetic PEGylation, therefore escaping the associated immunological liabilities (91, 92). This natural targeting capability is particularly relevant for ITP, because the therapeutic action must reach firstly the spleen which is the primary site of the autoantibody-mediated platelet destruction, and secondly the bone marrow, where an alteration is present in the niche, MSCs immunomodulation is compromised and the process of megakaryopoiesis is dysfunctional. Synthetic carriers may struggle to reach both the two main distinct compartments simultaneously, therefore EVs might be an excellent alternative to liposomes and previously implemented delivery platforms (93).

## 6 Clinical translation and ongoing trials

Cell- and cell-free based therapies represent potentially revolutionary approaches for a wide variety of pediatric diseases. One clinical trial is reported on ClinicalTrials.gov (NCT06813157) to be currently investigating MSCs in the treatment of refractory primary ITP, whereas no cell-free approaches, or therapies based on EVs currently in trial in pediatric ITP are registered. Nevertheless, regarding the novel application of EVs therapy in other pediatric diseases, as of the date of the manuscript (28 July 2026) 34 studies are ongoing as reported in ClinicalTrials.gov, filtering by the term “Extracellular Vesicles” in the selected range of age “Child” (from birth to 17 years), no further filters are implemented. Studies citing only Exosomes, Microvesicles or other sub-categories of EVs are specifically excluded as the purpose of the manuscript is to highlight the role of EVs as a whole. Therefore, the therapeutic application of EVs is still under evaluation and further studies are needed. **Figure 4** reports the different percentages of the 34 ongoing clinical trials mentioned, divided into areas of interest of the diseases under study.

**Figure 4:**
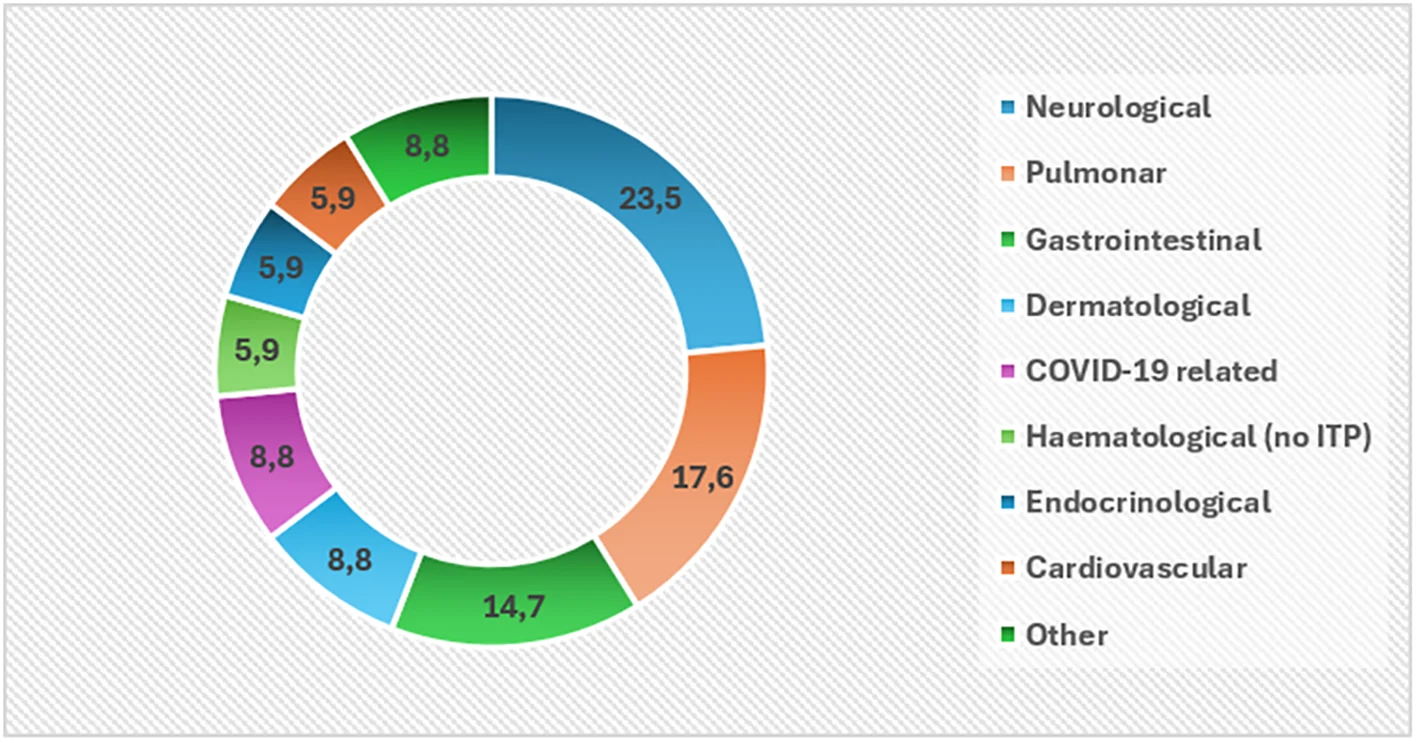
Clinical trials ongoing involving the use of EVs. Currently on the date of the manuscript (28 July 2026), 34 different studies are ongoing which revolve around the investigation of EVs in pediatric disorders, according to ClinicalTrials.gov. The trials have been divided into sub-categories depending on the disease area as described and the numbers in the figure represent the relative percentages. Trials were accounted for filtering per age “child” (from birth to 17 years-old) and additional term “Extracellular Vesicles”, excluding subcategories such as Microvesicles, exosomes, etc.

## 7 EVs limitations

Although EVs application is promising, clinical translation is still far away in the future. Despite these advances, a major hurdle remains the heterogeneity of engineered EVs, in fact even with advanced methods like electroporation, only a fraction of the total EV population may successfully incorporate the therapeutic payload while maintaining the correct size and surface markers. Furthermore, the scalability of these engineering processes must meet the rigorous GMP standards required for clinical translation in pediatric patients (94).

The route of EV administration is a critical determinant of biodistribution, therapeutic efficacy, and clinical feasibility but faces different limitations. Intravenous (IV) injection is the most commonly employed route in both preclinical and early clinical studies as it allows systemic distribution and exploits the natural homing and capability of EVs to reach specific tissues, including the spleen and bone marrow in ITP (95, 96). On the other hand, intraperitoneal (IP) administration has been widely used in murine models, offering high bioavailability and prolonged circulation, though its translational relevance to human patients remains limited (97, 98). Emerging evidence suggests that oral administration of EVs is feasible due to their resistance to gastrointestinal degradation, potentially enabling non-invasive delivery, although bioavailability remains a significant challenge (30, 99). The choice of administration route must be carefully considered in the context of ITP, where therapeutic EVs are required to reach both the spleen as the primary site of platelet destruction, and the bone marrow where megakaryopoiesis is impaired.

Another limitation is the difficulty in isolation and purification of EVs from biological fluids that can be achieved through several complementary methods, each possessing inherited advantages and limitations. Ultracentrifugation remains the most widely used technique, offering high yield but limited purity due to co-isolation of other molecules such as protein aggregates and non-vesicular particles (100). On the other hand, size-exclusion chromatography (SEC) provides superior separation based on hydrodynamic radius, yielding preparations with higher vesicular purity but lower recovery rates. Last but not least, tangential flow filtration (TFF) enables scalable, GMP-compliant processing suitable for clinical-grade production, while immunoaffinity capture using surface markers such as CD63 or CD81 allows isolation of specific EV subpopulations with high specificity but still at reduced throughput (101, 102). The latest MISEV2023 guidelines emphasize that the choice of isolation method directly influences the purity, heterogeneity, and functional characteristics of the resulting EVs, and recommend that studies explicitly report the isolation technique employed to ensure reproducibility (24, 94).

Regarding storage and stability, although evidence suggests that EVs might be stored at -80 °C for up to 6 months without loss in integrity or bioactivity, the long-term stability of MSC-derived EVs remains one of the critical concerns for clinical translation. Notably, lyophilization with trehalose as a cryoprotectant has shown promising results in preserving EVs function and morphology (94). Nowadays, a set protocol for storage for MSC-derived EVs is still not available, therefore establishing a standardized procedure under GMP is essential for clinical application and future therapeutic perspectives.

A further limitation that must be explicitly acknowledged concerns the dual nature of EVs, which may act simultaneously as therapeutic agents and as pathogenic mediators as reported in ITP. Plasma- and B-cell-derived EVs from ITP patients may transport pathogenic cargo which exacerbate the disease, including miR-363-3p, which impairs Treg-mediated immune suppression, and/or signaling that disrupts megakaryocyte apoptosis through Bcl-xL/caspase pathways (63, 64). This intrinsic duality represents the single largest translational risk of the platform, the risk of inoculating a deleterious payload rather than a beneficial one, therefore, without validated criteria to distinguish “therapeutic” from “pathogenic” cargo, EV preparations could inadvertently transfer molecules that sustain rather than resolve the autoimmune process. In the specific context of ITP, this distinction could be operationalized by benchmarking the cargo of MSC-EV preparations against the pathogenic EV signatures already characterized in patient plasma, such as the elevated miR-363-3p levels and the pro-apoptotic signaling described above, so that candidate therapeutic preparations are explicitly verified to be free of or de-enriched in these disease-associated molecules.

Although several issues hindering the clinical translation of EVs as therapeutic vehicles have been identified, addressing them will require a multi-layered validation framework.

Firstly, the composition of each EV preparation should be carefully defined rather than assumed, thus cargo identification must be established through molecular characterization, including transcriptomic and proteomic profiling (24, 103).

Secondly, a given EV preparation should produce replicable and validated immunomodulatory effects and functional potency assays must be performed to assess that this effect is stable across different administration routes, manufacturing platforms, and overcome heterogeneity of EVs from healthy donors to ensure identity, purity, sterility, potency and most importantly stability (104, 105).

Consequently, isolation and manufacturing must proceed under standardized, GMP-grade conditions that minimize co-isolation of non-EV contaminants and ensure batch-to-batch reproducibility, since cargo heterogeneity is strongly influenced by culture conditions and donor variability (24, 105). Only through the combination of these steps can the therapeutic cargo of MSC-EVs be prospectively distinguished from the disease-associated cargo carried by patient-derived EVs before clinical application.

### 7.1 Limitations of EVs-based therapy in pediatric ITP

Throughout this review we discussed the mechanistic and therapeutic evidence of EVs-based therapy in ITP, including the immunomodulatory roles of miR-146a-5p and miR-199a-5p and the involvement of the HMGB1-TLR4 pathway in MSCs dysfunction (48, 54, 57, 58). Although the studies previously described are reported as effective, they derive exclusively from adult patients or murine models, therefore no evidence on EVs-based therapy in pediatric ITP is currently available to our knowledge.

**Figure 3** represents the therapeutic rationale for MSC-EVs application in pediatric ITP, extrapolated and proposed by previous evidence in adult ITP and mouse models, therefore not the well-established mechanism of action. Pediatric ITP differs from the adult disease in several relevant respects, hence it is not trivial to highlight this distinction.

In fact, pediatric ITP includes a higher rate of spontaneous remission, a distinct temporal pattern of chronicity, a different impact on an immune system that is still developing, and a treatment landscape in which aggressive second-line options are deliberately delayed in favour of less invasive therapies (106–108). The bone marrow niche itself is a dynamic microenvironment whose cellular composition and cytokine milieu may differ between growing patients and adults, potentially influencing both MSCs behavior and EVs-mediated communication. Consequently, the magnitude and quality of the immunomodulatory response elicited by MSC-EVs in children cannot be assumed to mirror that observed in adults, and factors such as EVs dosing, source of MSCs (allogeneic versus autologous), donor variability, and the timing of administration within the disease course remain entirely undefined in the pediatric setting (50).

Bridging this gap will require dedicated studies in pediatric ITP cohorts or age-appropriate disease models, standardized characterization of EVs cargo in pediatric biological samples, and ultimately controlled clinical trials. Until such evidence becomes available, MSC-EVs should be regarded as a promising but not yet validated therapeutic strategy for pediatric ITP.

## 8 Potential developments and future perspectives

Extracellular vesicles represent a novel and promising therapeutic approach for pediatric ITP as their unique biological properties like low immunogenicity, inherent biocompatibility, natural capability to traverse biological barriers, and ability to interact simultaneously with different targets to modulate the immune response, position them currently as excellent candidates for future applications, and superior to both conventional pharmacological approaches and other current synthetic nanocarriers. MSCs-derived EVs in particular act concurrently to restore T-cell balance, promote megakaryopoiesis, and re-establish the bone marrow niche, addressing the complex, multi-layered pathophysiology of ITP in a way that no current therapy achieves.

However, significant challenges remain before this potential therapeutic application may be translated into clinical practice. In fact, the major limitations in EVs therapeutic application derive mainly from the heterogeneity of EV populations, the difficulty of achieving reproducible large-scale production and isolation methods under GMP conditions. In particular, the absence of standardized cargo-loading protocols and the lack of validated quality-control biomarkers, together with complex pharmacology and pharmacodynamics analysis contribute to the difficulties in developing viable EVs-based therapies not only regarding pediatric patients, but for any clinical application. Before clinical application is implemented, all previously mentioned limitations represent substantial barriers to clinical translation that must be resolved and investigated (109, 110).

As discussed, the dual nature of EVs as both therapeutic vehicles and disease mediators where endogenous disease-derived vesicles may themselves contribute to ITP pathogenesis, adds an additional layer of complexity that demands strict characterization of therapeutic EVs cargo, at the moment not yet available. Furthermore, regulatory frameworks for EVs-based therapeutics are still in their preliminary stage, and no guidelines specific to extracellular vesicle drugs have yet been issued by major regulatory authorities as the field is still investigating and expanding. Future research should prioritise the three major limitations leading to the development of standardized application of EVs-based therapies, thereby highlighting the need for scalable manufacturing platforms capable of producing clinical-grade MSCs-EVs (no heterogeneity), the identification and validation of specific miRNA and protein cargo signatures that correlate with therapeutic efficacy in ITP (prognostic and disease biomarkers), and eventually, the design of adequately randomized clinical trials that can definitively establish the safety and efficacy of EVs-based therapies in pediatric populations.

Only through rigorous, multicenter clinical investigation can the promising application of EVs-based therapy be translated into a tangible and viable therapeutic option for children with immune thrombocytopenia.
